# Supplementary material for: Content-rich biological network constructed by mining PubMed abstracts
Source: BMC Bioinformatics. 2004 Oct 8;5:147. doi: 10.1186/1471-2105-5-147 (PMC528731; doi:10.1186/1471-2105-5-147)
Supplement: Additional File 2 — The original results of the above study (non-essential files are deleted to keep the file size under the limit set by BMC bioinformatics). [file 1471-2105-5-147-S2.bz2 › chilibotAdditionalFile2/dip05/635ID10933786E11022/html/B2M_FCGRT.html]

 


 **B2M** and **FCGRT** 
  
Found 24 abstracts in PubMed, retrieved 05.  
 

 What does Google say? 
 PDF only 
| .edu only 

---

**Interactive relationship** (e.g. stimulation, inhibition, etc)

**Neutral relationship**- Several functions attributed to FcRn  [ **FCGRT** ]  are affected in beta 2 microglobulin  [ **B2M** ]  beta 2 m deficient mice, suggesting that the alpha chain needs to assemble with beta 2 m to form a functional receptor.  Ref: 12006623 J Cell Sci, 2002
- The heavy chain HC of the neonatal Fc receptor  [ **FCGRT** ]  FcRn  [ **FCGRT** ]  for IgG is non convalently associated with beta 2 microglobulin  [ **B2M** ]  beta 2 m.  Ref: 12162790 Biochem J, 2002
- FcRn  [ **FCGRT** ]  is a heterodimer of an alpha chain and beta 2 microglobulin  [ **B2M** ]  beta 2 m and differs from other IgG Fc receptors in that it is structurally related to MHC class I molecules.  Ref: 12006623 J Cell Sci, 2002
- Functional reconstitution of human FcRn  [ **FCGRT** ]  in Madin Darby canine kidney cells requires co expressed human beta 2 microglobulin  [ **B2M** ] .  Ref: 12023961 J Biol Chem, 2002
- The neonatal IgG transporter FcRn  [ **FCGRT** ]  consists of two chains, FcRn  [ **FCGRT** ]  alpha and beta also known as beta 2 microglobulin  [ **B2M** ] , and is involved in transferring IgG molecules across both mammary and intestinal epithelial cells.  Ref: 12531282 Mol Immunol, 2003
- beta 2 Microglobulin  [ **B2M** ]  is important for cell surface expression and pH dependent IgG binding of human FcRn  [ **FCGRT** ] .  Ref: 12006623 J Cell Sci, 2002
- The major histocompatibility complex class I related neonatal Fc receptor  [ **FCGRT** ] , FcRn  [ **FCGRT** ] , assembles as a heterodimer consisting of a heavy chain and beta 2 microglobulin  [ **B2M** ]  beta 2 m, which is essential for FcRn  [ **FCGRT** ]  function.  Ref: 12023961 J Biol Chem, 2002

**Non-interactive relationship** (e.g. studied together, co-existance, homology, etc.)

- Using in situ hybridisation, the FcRn  [ **FCGRT** ]  alpha and beta 2 microglobulin  [ **B2M** ]  transcripts were localised to the epithelial cells of the PY intestine.  Ref: 12531282 Mol Immunol, 2003
- The results are consistent with the hypothesis that changes in FcRn  [ **FCGRT** ]  alpha and beta 2 microglobulin  [ **B2M** ]  gene expression take place in the possum PY intestine to accommodate changes in maternal milk composition to meet the changing immunological demands of the PY.  Ref: 12531282 Mol Immunol, 2003
- Absence of TPKTSVT placental homing in mice lacking beta 2 microglobulin  [ **B2M** ]  beta 2 m suggests FcRn  [ **FCGRT** ]  beta 2 m as a target for the TPKTSVT, which is unexpected, given the normal development of FcRn  [ **FCGRT** ]  beta 2 m deficient progeny.  Ref: 12242328 Proc Natl Acad Sci U S A, 2002
- However, no prolactin receptor transcripts were detected in the same epithelial cells suggesting that the observed changes in FcRn  [ **FCGRT** ]  alpha and beta 2 microglobulin  [ **B2M** ]  gene expression in the proximal intestine are not modulated directly by prolactin.  Ref: 12531282 Mol Immunol, 2003
- Genomic sequences located 5 to the start of transcription of the FcRn  [ **FCGRT** ]  alpha and beta 2 microglobulin  [ **B2M** ]  genes were cloned and analysed for predicted cis acting transcription control elements.  Ref: 12531282 Mol Immunol, 2003
- Both the FcRn  [ **FCGRT** ]  alpha and beta 2 microglobulin  [ **B2M** ]  genomic sequences contained STAT5 binding motifs consistent with the transcription of both genes being modulated by prolactin.  Ref: 12531282 Mol Immunol, 2003
